# Audit of Shared Care Guideline Compliance for ADHD Patients: Monitoring Physical Observations by General Practitioners

**DOI:** 10.1192/bjo.2024.642

**Published:** 2024-08-01

**Authors:** Chinwe Utomi

**Affiliations:** Leeds and York Partnership NHS Trust, Leeds, United Kingdom

## Abstract

**Aims:**

To evaluate the adherence to shared care guidelines for ADHD patients by assessing if their Blood Pressure, Pulse Rate, and Weight have been monitored at least twice within the last year by their GP, as recommended by NICE guidelines.

Following initiation and stabilization on an ADHD medication, shared care with the GP is initiated whereby the GP is responsible for prescribing the medication, and monitoring physical observations every 6 months.

**Methods:**

Data Collection:

1. a. Collate the list of patients that were due for annual structured review in August and September 2023 from the team's shared drive.
2. b. Randomly select 50 patients from this list.

Inclusion criteria:

1. Patient must have been on ADHD medication in the past 12 months.

Data Assessment:

1. a. Access the GP records (Patient Practice Management system) for the selected 50 patients.
2. b. Review the patient records for each of these 50 patients to identify when Blood Pressure, Pulse Rate, and Weight measurements were recorded within the last 12 months.
3. c. Record the date and results of the Blood Pressure, Pulse Rate, and Weight measurements for each patient.
4. d. Determine if each patient had these measurements done at least twice within the last year as per NICE guidelines.
5. e. Calculate the percentage of patients who met this guideline.

**Results:**

Sample size: 22 patients patients met the inclusion criteria.

- Blood pressure checked within the last 6 months – 22/22 (100%)
  Blood pressure checked within the last 1 year – 19/22 (86%)
- Pulse rate checked within the last 6 months – 20/22 (90%)
  Pulse rate checked within the last 1 year – 18/22 (81%)
- Weight checked within the last 6 months – 21/22 (95%)
  Weight checked within the last 1 year – 20/22 (90%)

8 out of 22 had a “significant” change in their BP reading.

This significance is in keeping with NICE Guidelines that is, an increase of 2–4 mmHg for patients on ADHD medication, but this is generally not significant in terms of risk.

**How was the project outcome disseminated?**

A letter was sent out to GP practices commending the positive outcome of the audit. Recommendations for further improvement were suggested flagging up a review if there is a reduction of 10% or more in body weight within 12 months of treatment.

**Conclusion:**

The positive outcome of the audit shows the effectiveness of current practices. However, it's important to maintain a commitment to ongoing improvement. Regular evaluations and audits remain essential to adapt to evolving guidelines, address emerging challenges, and sustain a culture of excellence in healthcare delivery.

